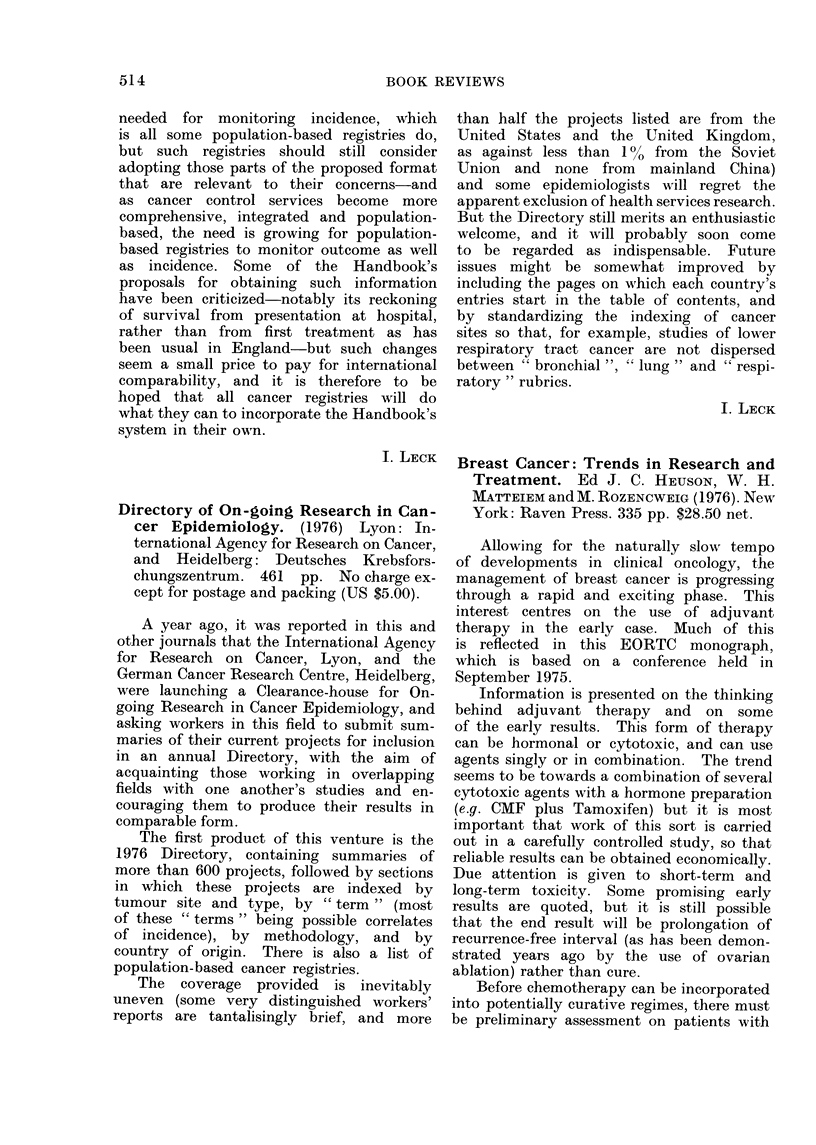# Directory of On-going Research in Cancer Epidemiology

**Published:** 1977-04

**Authors:** I. Leck


I. Leck

**Directory of On-going Research in Cancer Epidemiology.** (1976) Lyon: International Agency for Research on Cancer, and Heidelberg: Deutsches Krebsforschungszentrum. 461 pp. No charge except for postage and packing (US $5.00).

A year ago, it was reported in this and other journals that the International Agency for Research on Cancer, Lyon, and the German Cancer Research Centre, Heidelberg, were launching a Clearance-house for On-going Research in Cancer Epidemiology, and asking workers in this field to submit summaries of their current projects for inclusion in an annual Directory, with the aim of acquainting those working in overlapping fields with one another's studies and encouraging them to produce their results in comparable form.

The first product of this venture is the 1976 Directory, containing summaries of more than 600 projects, followed by sections in which these projects are indexed by tumour site and type, by " term " (most of these " terms " being possible correlates of incidence), by methodology, and by country of origin. There is also a list of population-based cancer registries.

The coverage provided is inevitably uneven (some very distinguished workers' reports are tantalisingly brief, and more than half the projects listed are from the United States and the United Kingdom, as against less than 1% from the Soviet Union and none from mainland China) and some epidemiologists will regret the apparent exclusion of health services research. But the Directory still merits an enthusiastic welcome, and it will probably soon come to be regarded as indispensable. Future issues might be somewhat improved by including the pages on which each country's entries start in the table of contents, and by standardizing the indexing of cancer sites so that, for example, studies of lower respiratory tract cancer are not dispersed between " bronchial ", " lung " and " respiratory " rubrics.

I. Leck